# Nontargeted metabolomics study and pharmacodynamic evaluation of bidirectional fermentation for *Ganoderma lucidum* with *Marsdenia tenacissima*

**DOI:** 10.3389/fphar.2022.1012063

**Published:** 2022-10-26

**Authors:** Runtian Li, Zhiguang Zhang, Xinxin Su, Jiaoneng Yu, Lin Lu, Tongxiang Liu

**Affiliations:** ^1^ School of Pharmacy, Minzu University of China, Beijing, China; ^2^ Key Laboratory of Ethnomedicine (Minzu University of China), Ministry of Education, Beijing, China

**Keywords:** *Marsdenia tenacissima*, *Ganoderma lucidum*, fermentation, non-target metabolomics, C_21_ steroidal glycosides, pharmacodynamic

## Abstract

Lung cancer is one of the malignant tumors with the fastest incidence rate and mortality growth and the greatest threat to human health and life. *Marsdenia tenacissima* is an antitumor of Chinese medicine. However, *Marsdenia tenacissima* has low bioavailability in the human body and most of its main active substances are aglycones, such as Tenacigenin A, Tenacigenin B. This study aims to produce biotransformation products rich in pungent saponins by using *Marsdenia tenacissima* as a fermentation medium of *Ganoderma lucidum*. Non-targeted metabolomics analysis was carried out on the fermentation products after the optimization process. A total of 249 differential metabolites were detected, and the content of saponins increased from 0.1% to 0.41% and most of them were tenacigenin. Furthermore, the biotransformation of C_21_ steroidal glycosides in *Marsdenia tenacissima* was the central reaction in this fermentation process. Pharmacodynamics resewed that the anticancer effect of *Marsdenia tenacissima* was significantly enhanced after fermentation, mainly through inhibiting the growth and apoptosis of cancer cells.

## 1 Introduction

Since ancient times, the Chinese have used microbial fermentation to process traditional herbal medicines. From the time of the Han and Jin dynasties, fermentation technology was applied to Chinese medicine processing, when koji was added to different drugs to make various medicinal Yaoqu (Chen, 2010). Modern Chinese medicine fermentation combines traditional methods with modern microecology, bioengineering, fermentation engineering, and other techniques, also known as biotransformation technology that enhances its original characteristics and produces new effects based on the action of microorganisms under appropriate temperature, humidity, and moisture conditions. It expands its application scope to meet clinical needs (Cai et al., 2012; Li et al., 2020).

*Ganoderma lucidum* (Fr.) Karst has been used in Asian countries such as China, Japan and South Korea for more than 2,000 years. It can be eaten and used as medicine. It strengthens the spirit, promotes blood circulation and menstruation, prolongs life, beauty, and so on (Chan et al., 2021). The beneficial health effects of *G. lucidum* might be attributed to bioactive compounds such as polysaccharides and terpenoids (Benkeblia, 2016). Besides the health effects, *G. lucidum* can also degrade lignins, cellulose and hemicellulose, owing to its ability to synthesize relevant enzymes such as lignin-modifying enzymes (Postemsky et al., 2017).

*Marsdenia tenacissima* (Roxb.) Moon [Apocynaceae; Marsdeniae tenacissimae caulis] as traditional Chinese medicine, is mainly produced in Yunnan Province of China. Pakistan, India, Indonesia and Sri Lanka are also distributed. It relieves cough and asthma, eliminates phlegm, and removes heat and detoxifies (Xing et al., 2003; Wang P. et al., 2018). In modern research, *Marsdenia tenacissima* has been proved to have effects of anticancer, anti-asthma, bacteriostasis, liver protection, diuresis and immune regulation (Wang et al., 2014; Haiming et al., 2016). Its preparation XiaoAiPing injection has been widely used in the clinic and achieved a good therapeutic effect (Shi et al., 2019). Among these bioactive compounds, saponins from *Marsdenia tenacissima* were highly investigated. More than 100 saponins from *Marsdenia tenacissima* have been identified and can be divided into two major types: 1) C_21_ steroidal glycosides in *Marsdenia tenacissima* includes tenacissoside A-P, marsdenoside A-M, tenacigenoside A-L, tenacigenin A-D and so on. 2) Pentacyclic triterpenes include Oleanolic acid, and Ursolic Acid (Yin, 2019). The main active components are C_21_ steroidal glycosides which has low bioavailability but can be converted into tenacigenin by gastrointestinal microorganisms. Many studies indicated that the anticancer activity of petroleum ether extracts higher than micropolar molecules from *Marsdenia tenacissima* (Hu et al., 2020). Hence, it is advantageous to convert the significant polysaccharide of saponins from *Marsdenia tenacissima* into another minor aglycone of *Marsdenia tenacissima* with higher bioavailability and bioactivities. Therefore, research efforts must focus on transforming macropolar to another low polarity. Microbial transformation is more advantageous because of its low cost, and eco-friendliness and it can be scaled up with good reproducibility. Therefore, microbial transformation is most desirable for industrial applications (Han et al., 2007).

With the development of technologies based on nuclear magnetic resonance, mass spectrometry, or chromatography, a great deal of information about metabolites and multivariate statistical tools have been collected and developed (Jia et al., 2022). A rapid automatic analysis and identification system combined with simple operating methods offered new opportunities to globally analyze all small-molecule metabolites present within fungi in a particular physiological state (Gika et al., 2019). Some substances of traditional *Marsdenia tenacissima* may also promote the growth and metabolism of medicinal *Ganoderma lucidum* and the production of active ingredients. Ganoderma lucidum may also change its metabolic pathways in the particular environment of *Marsdenia tenacissima* with *Ganoderma lucidum* and compound prescriptions, thus forming new active ingredients or changing the relationship between active ingredients (Sadiq et al., 2021). This study focused on the biotransformation of *Marsdenia tenacissima* with the help of *Ganoderma lucidum* enzyme system, with the aim of co-fermenting *Marsdenia tenacissima* with *Ganoderma lucidum* in culture. The *Marsdenia tenacissima*, *Ganoderma lucidum* and *Marsdenia tenacissima* with *Ganoderma lucidum* co-fermentation were analyzed by untargeted metabolomics methods. Chemometric data analysis of polysaccharides, saponins, organic acids, alkaloids and flavonoids were performed to precisely identify different parts of *Marsdenia tenacissima* with *Ganoderma lucidum* co-fermentation. Because fermentation is influenced by various factors, the response surface methodology (RSM) was applied to investigate the final conditions of fermentation to support the ability to expand production in the future.

## 2 Materials and methods

### 2.1 Materials

#### 2.1.1 Medicinal materials and fungi

*Marsdenia tenacissima* (MT) was collected from Kunming, Yunnan province. The samples were identified by Professor Tongxiang-Liu and kept at School of Pharmacy of Minzu University of China. MT was extracted by hot water (100°C, MT/water = 1/10, w/v) for 30 min and the MT extraction residue was collected as the material. The *Ganoderma lucidum* (GL) was purchased by BeNa Culture Collection (NO31732, Xinyang City, Henan Province, Chin). The HPLC-grade solvents acetonitrile and methyl alcohol were purchased from Thermo (United States).

#### 2.1.2 Cell lines and cultures

The LLC Mouse lung cancer cell were used for *in vivo* assessment. The LLC Mouse lung cancer cell line was purchased from the National Infrastructure of Cell Line Resource (Beijing, China), were maintained in DMEM supplemented with 10% fetal bovine serum, 100 U/ml penicillin, and 100 μg/ml streptomycin in a humidified atmosphere at 37°C under 5% CO_2_.

#### 2.1.3 Animals

Male C57BL/6J mice aged 6–8 weeks and weighing 19 ± 2 g were obtained from Vital River Laboratory Animal Technology Co., Ltd. (Beijing, China). The animals were kept in an environmentally controlled room with a 12/12 h light/dark cycle and unlimited access to food and water. They were disinfected regularly. This study was conducted by the recommendations of the experimental animal center of Minzu University (No.ECMUC2019008AA). The protocol was approved by the Experimental Animal Ethical Committee of Minzu University.

### 2.2 Culture condition of the LAB strains

The GL were evenly spread on a potato dextrose agar (PDA) plate and cultured at 28°C–30°C for 5–6 days until mature spores formed. Then, we added sterile water to dilute the spore suspension to a concentration of 106–107 units/ml. We inoculated 2.0 ml of spore suspension in 50 ml of LB broth (AOBOXING BIO-TECH CO.,LTD, Beijing, China) and cultured the fungi at 120 rpm and 28°C for 1–2 days to obtain the seed solutions.

### 2.3 Fermentation of MT with GL

The seed solution was seeded in an aqueous solution containing only MT after sterilization, according to Supplementary Table S1, at 28°C and 120 rpm for 744 h. The sample were filtered and centrifuged to get mycelium. The myceliums were dried at 100°C, and the dry matter was pulverized and weighted.

### 2.4 Fermentation method

#### 2.4.1 One-factor-at-a-time experiment

One-factor-at-a-time experiment was used to investigate the effects of fermentation parameters on production. Six process parameters including fermentation time (2–10 days), fermentation temperature (18, 23, 28, 33, 38°C), inoculation volume (2%, 4%, 8%, 10%, 14%), MT concentration (70, 90, 110, 130, 150 mg/L), speed of shaking machine (80, 120, 160, 200, 240 rpm) and initial pH of media (4.0, 5.0, 6.0, 7.0, 8.0) were routinely investigated in 250 ml shake flask. If not specified, the fermentation temperature was 28°C, the inoculum was 6%, the fermentation time was 6 days, the MT concentration was 90 mg/L, the pH was 6.0 and the speed was 120 rpm (each group of six samples).

#### 2.4.2 Response surface methodology experimental design

In this study, The RSM experiment was composed of four factors and three levels and the yield of dry mycelium was set as an objective function. The pH was 5. 8 of fermentation and the temperature was 28°C of fermentation, according to the result of the one-factor-at-a-time experiment. The effects of fermentation parameters such as fermentation time, MT concentration and speed of shaking machine were evaluated. The factors and the levels used in this study are depicted in Supplementary Table S1. The complete design consisted of 29 combinations, including five replicates of the center point (Supplementary Table S2). The response function (Y) was partitioned into linear, quadratic and interactive components:

$$Y=b_{0}+\overset{k}{\underset{i}{\sum }}b_{i}X_{i}+\overset{k}{\underset{i}{\sum }}b_{ii}X_{i}^{2}+\overset{k}{\underset{i>j}{\sum }}b_{ij}X_{i}X_{j}$$

The polynomial coefficients were composed of b_0_ (constant term), b_1_ b_2_ b_3_ and b_4_ (linear effect), b_12_ b_13_ b_14_ b_23_ b_24_ and b_34_ (quadratic effects), b_11_ b_22_ b_33_ and b_44_ (interaction effects). The variance analysis was generated and the effects and regression coefficients of each linear, quadratic and interactive term were determined (Supplementary Table S2). Then the regression coefficient is used for the statistical calculation to generate contour map from the regression model (Berengut, 2006).

Design Expert 10.0 software was used to design the experiments for interaction studies, and the experimental design was represented in Supplementary Table S2. The purpose of this study was to improve the yield of fermentation, so the parameters combined with the highest yield was the optimum parameters combination. The experiment design and analysis of variance were performed by statistical software Design Expert 10.0.

### 2.5 Metabolomics experiments

#### 2.5.1 Sample preparation and LC-MS analysis

10 mg *Marsdenia tenacissima* (MT), *Ganoderma lucidum* (GL) and *Marsdenia tenacissima* with *Ganoderma lucidum* co-fermentation (MGF) were extracted by 500 μl methanol: H_2_O (7:3), shaked at 4°C for 1 min, sonicated 30 min and centrifuged for 20 min at 1,200 rpm. The supernatants were collected, filtered through 0.22 μm polyvinylidene fluoride membrane, and added to the LC-MS/MS.

To identify the metabolite from MT, GL and MGF, three samples from three independently biological replicates were randomly analyzed by LC-MS/MS analysis. The extracts were analyzed by ExionL C (AB Sciex, Ontario, Canada) coupled to a SCIEX Triple TOF 5600 + mass spectrometer, operating in both positive and negative ionization mode. Chromatographic separation of samples was performed using an ACQUITY UPLC HSS T3 (2.1 × 100 mm, 1.8 μm). The column temperature was maintained at 40°C. Mobile phase A was formic acid (0.1%) (v/v), and B was acetonitrile containing 0.1% formic acid. The elution profile was set as following:0 min 5%B; 2 min 5%B; 14 min 98% B; 17 min 98% B; 17.1 min 5% B; 20 min 5%, flow rate 0.3 ml/min.

#### 2.5.2 Mass spectrometry conditions

In the negative (positive) ion source acquisition mode, IDA scanning modes were high sensitivity, and the dynamic background was deducted, ion source parameters set to sheath gas flow rate 35, Gas Ⅰ flow rate 55, Gas Ⅱ flow rate 55, temperature 550°C, spray voltage −4,500 V (positive spray voltage 5,500 V). The scan time of 20 min, the primary scan range of 100–1,200 m/z, each primary scan was followed by 12 secondary scans, secondary scanning The range of the primary scans was 100–1,200 m/z, each primary scan was followed by 12 secondary scans, the secondary scan range was 50–1,200 m/z, the secondary accumulation time was 0.05 s, the collision energy was −40 neV, and the collision energy range was theoretical frequency ±20.

#### 2.5.3 On-machine sample quality control

The quality control sample (QC) was prepared by mixing MT, GL and MGF extract in equal volume, and three replicates were named QC_1_, QC_2_ and QC_3_, respectively. The processing and detection methods of quality control and analysis samples were the same. One QC was inserted between every three samples to ensure the instrument’s stability during the injection process.

### 2.6 Antitumor activity *in vivo*

Seventy mice were divided into seven groups, the model group, the control group, the MT group, the MGF (high) group, the MGF (medium) group, the MGF (low) group and the cisplatin group. Randomly take ten as a control group, and another sixty mice were subcutaneously injected with LLC cells (1.0 × 10^6^ cells in a volume of 0.2 ml PBS per mouse) on the right flank. After 6 days, the successful establishment of the model was confirmed by touching the tumor nodules (about 5 mm). The corresponding doses of MT and MGF (medium) were administered as crude drug according to Chinese Pharmacopoeia 2020. The details are as follows: In adults weighing 60 kg, human-equivalent dose (HED, mg/kg) = Mouse dose (mg/kg) × (Mouse Km/Human Km) [Km: the correction factor is estimated by dividing the average body weight (kg) of species to its body surface area (m^2^)]. According to the HED, we calculated the dose for mouse, as the dose of MT was 4.11 g/kg (crude drug/body weight, equivalent to the extract of this experiment 3.16 g/mg). The dose of MGF were the amount of fermentation product converted into the MT original medicinal material. Tumor-bearing mice were randomly grouped (n = 10 per group) in the model group, MGF (medium) group (20.50 g/kg, MT crude drug/body weight), MGF (high) group (40.11 g/kg, MT crude drug/body weight), MGF (low) group (10.25 g/kg, MT crude drug/body weight), MT group (20.50 g/kg, MT crude drug/body weight) and cisplatin group (positive control, 200 mg/kg). Model group and blank group mice normal saline 0.4 ml/d with gastric lavage; the mice in the cisplatin group were intraperitoneally injected with 0.4 ml of cisplatin injection (0.2 mg/ml) twice a week for 4 times. The MGF group was given 0.4 ml/d, with gastric lavage, and the MT group was given 0.4 ml/d, solution of fermentation products of MT and Ganoderma lucidum, with gastric lavage, and the MT group was given 0.4 ml/d of MT extract, with gastric lavage. After 14 days of treatment, mice were euthanasia treated, the tumor tissue and lung tissues were collected.

### 2.7 Western blotting

For immunoblot analysis, lung cancer tissue was harvested and lysed. Protein concentrations were determined using the BCA protein assay kit (Beyotime, Shanghai, China). Protein samples were separated by 10% SDS-PAGE and blotted onto PVDF membranes. p-38 MAPK, mTOR, caspase-9 and caspase-3 were used to detect the corresponding proteins (The information of antibody in Supplementary Table S7). After washing, the membranes were incubated with appropriate horseradish peroxidase-conjugated secondary antibodies (Beyotime, Shanghai, China). Protein bands were visualized using ECL chemiluminescence kit (Beyotime, Shanghai, China).

### 2.8 Statistical analyses

All the fermentation experiments were repeated for six times and expressed by the ±SD (standard deviation) of the mean. In the one-factor-at-a-time experiment, One-way ANOVA was performed using SPSS (IBM Corporation, Version 25.0) software and a *t*-test was used for statistical significance (*0.01 ≤ *p* ≤ 0.05, **0.001 ≤ *p* ≤ 0.01, ***p ≤ 0.001). The response surface experiment was analyzed by Design-Expert (6.0.10 Trial, elaware, United States Echip, 1993) software.

Using SCIEX OS software to identify metabolites is mainly based on Mass Error, Retention Time, Isotope Match, MS/MSLibrary Purity Score and Computational Molecular Formula. The library settings parameter is Mass Error < 5ppm, Isotope Ratio Difference <20 and Library Score >50. Using R (Version3.6.2) language to draw clustering heatmap, principal component analysis (PCA) and orthogonal partial least-squares describe mination analysis (OPLS-DA) were performed by SIMCA software (v13.0, Switzerland). The selection of differentially expressed metabolites in MT, GL and MGF was based on a statistically significant threshold of variable influence on projection (VIP) values and *p*-values obtained from double-tailed *t*-test on the normalized peak areas. R^2^Y and Q^2^ explainable model variables and model predictability and the advantages or disadvantages of the model can be distinguished. The database selected in this experiment was TCM MS/MS Library 2.1 database, and the metabolites with CV < 30%, VIP >1 and *p*-value < 0.05 in QC samples were selected as differential metabolites.

## 3 Results

### 3.1 One-factor-at-a-time experiment

**FIGURE 1:**
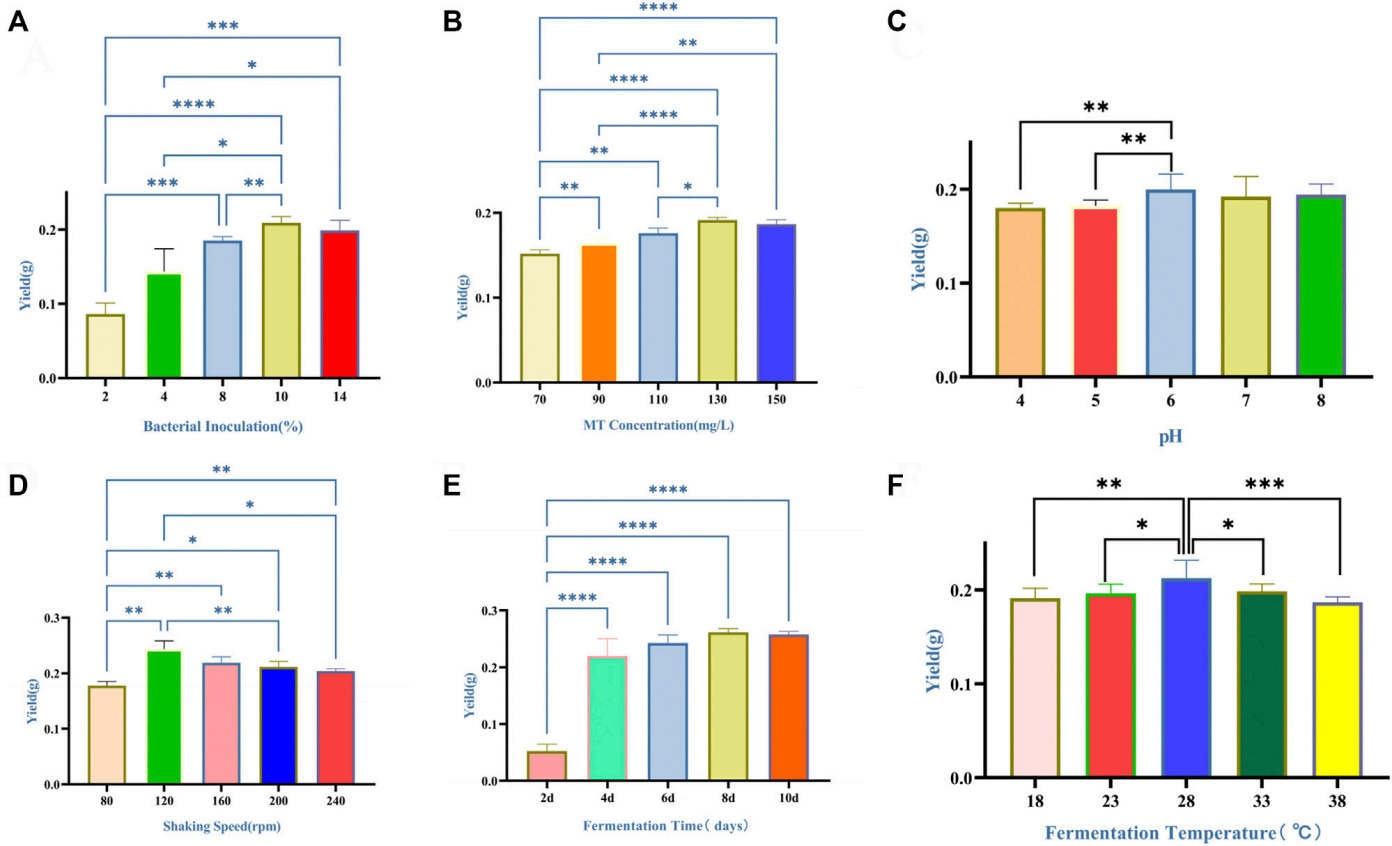
Effects of six fermentation factors on production of the yield of dry mycelium of *Marsdenia tenacissima* with *Ganoderma lucidum* co-fermentation **(A–F)**. Data were expressed as means ± SD (n = 6). *p ≤ 0.05; **p ≤ 0.01; ***p ≤ 0.001; ****p ≤ 0.0001. **(A)** Bacterial inoculation. **(B)** MT concentration. Data *versus* 70 mg/L of MT concentration. **(C)** pH. **(D)** Shaking speed. **(E)** Temperature. **(F)** Fermentation time.

The effects of six fermentation parameters on the yield of dry mycelium were show in Figure 1. As shown in Figure 1A, the yield of dry mycelium increased by the bacterial inoculation from 2% to 8% and reached the peak value of 8%–10%. The yield of dry mycelium increased with the MT concentration, and stopped increasing when the MT concentration was 130 mg/L (Figure 1B). The difference in yield of dry mycelium obtained was not significant with the pH increased from 4 to 8 (Figure 1C). When the shaking speed was 120 rpm, the yield of dry mycelium reached peak value (Figure 1D). As shown in Figure 1E, the optimum fermentation temperature was at 28°C for the production of the yield of dry mycelium. The yield of dry mycelium increased from 2 to 8 days and reached the peak value on the 8th–10th day (Figure 1F).

### 3.2 Response surface methodology experiment

#### 3.2.1 Statistical analysis

The experimental values for the yield of dry mycelium are presented in Supplementary Table S1. The regression coefficients for the second-order polynomial equations and results for the linear, quadratic and interaction terms are presented in Supplementary Table S2. The statistical analysis indicates that the *p* < 0.001 of the regression model and the lack of fit *p* = 0.263 which was not significant in Supplementary Table S2, indicates that the yield of dry mycelium by fermentation is adequately fitted to the actual situation. The *R* ^2^ was 0.9185 in Supplementary Table S2, the better the empirical model fits the actual data.

#### 3.2.2 Effects of MT concentration, fermentation time, bacterial inoculation and shaking speed

**FIGURE 2:**
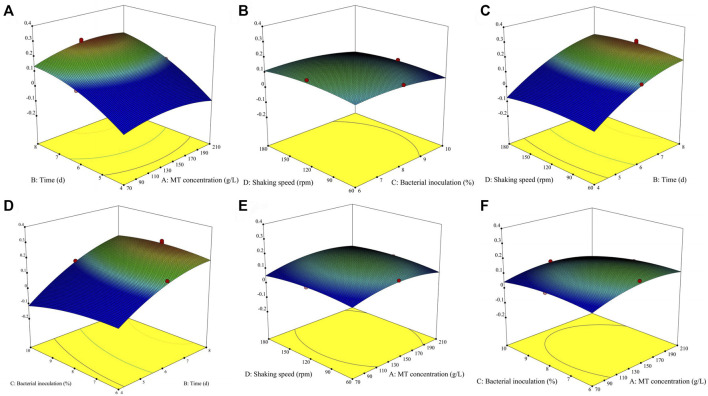
Graphical results in terms of the response surfaces of the format value of Y1 (g) of the yield of dry mycelium (Supplementary Table S2) from system optimizations. Joint graphical 3D analysis as a function of each of the variables involved [Part 1 **(A–F)**].

**FIGURE 3:**
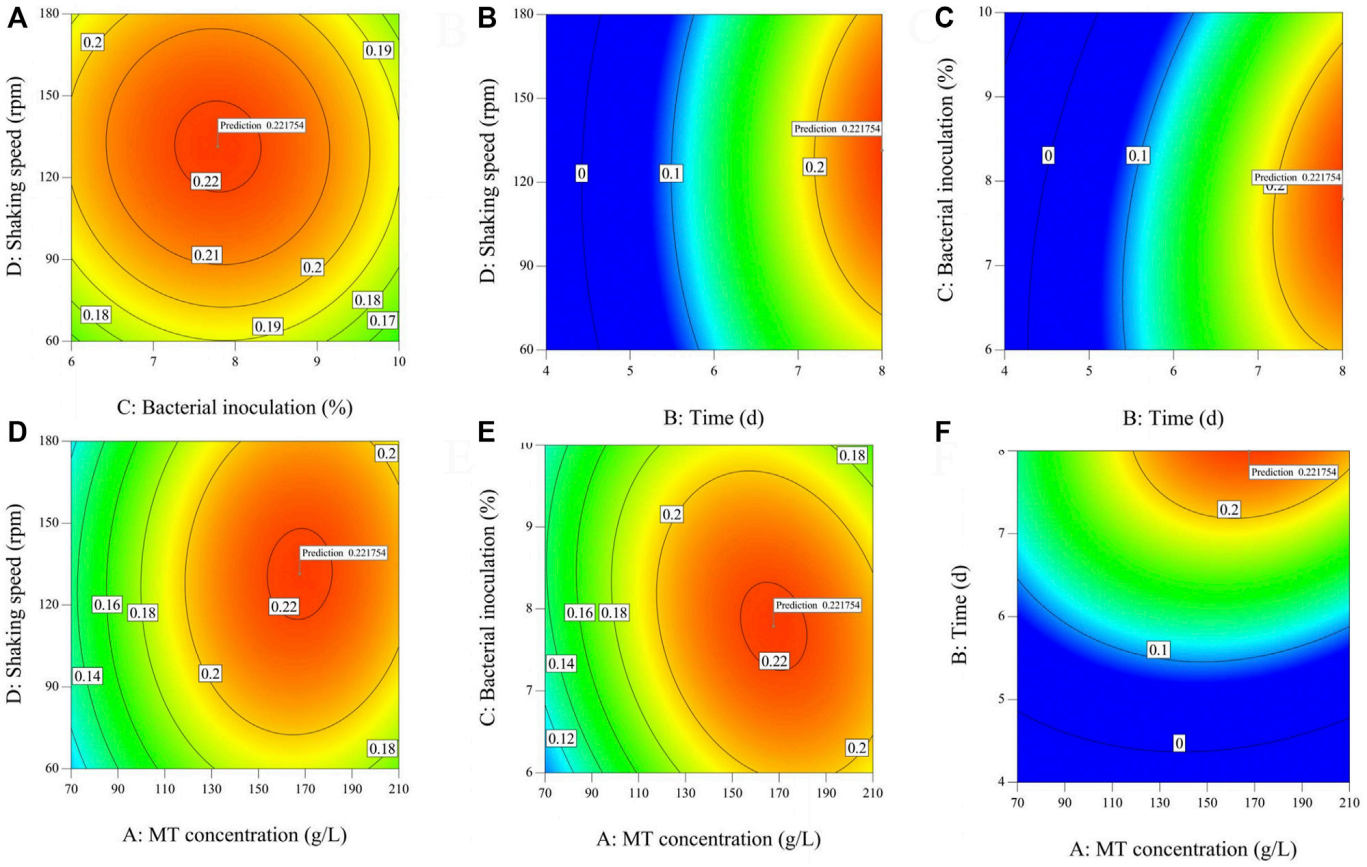
Contour of two factors influences each other [Part 2 **(A–F)**].

The effect of different fermentation treatment conditions on the yield of dry mycelium are reported (Supplementary Table S1) by the coefficient of the second-order polynomials. To aid visualization, the response surfaces for the yield of dry mycelium in Figure 2. Then Figures 2, 3 was divided into two subsections (1 and 2). As shown in Supplementary Table S2, the yield of dry mycelium mainly depended on fermentation time which linear effects (*p* < 0.01) and quadratic effects (*p* < 0.01) were significantly giving an overall curvilinear effect. There is also a significant relationship between dry weight yield of mycelium and quadratic terms (*p* < 0.01) of other factors resulting in a decrease with the increase of factors (Figure 2). It can be seen from Supplementary Table S2 that the linear effects of MT concentration (*p* > 0.05) and speed of the shaker (*p* > 0.05) are not obvious, so it has little effect on the fermentation experiment. It can also be found from Figure 2 that with the change of the MT concentration or rotation speed to be worn, there is no significant change in the product obtained by fermentation. The quadratic effect of fermentation time (b_22_ < 0) and linear effects (b_2_ > 0), As a result, the yield of dry mycelium products will gradually decrease after reaching a certain value over time (Figures 2A–C). Because the linear effect of bacterial inoculation (b_3_ < 0) was negatively associated, the yield of dry mycelium decreases with the increase of bacterial inoculation (Figures 1D,E). The contour shows the optimum conditions of the fermentation (Fan et al., 2008). The maximum value obtained by the response surface falls in the minimum ellipse in the contour map, and the contour map is elliptical. The optimal conditions affecting fermentation were analyzed and the maximum yield was predicted to be 0.221754 g under these conditions (Figure 3 contour).

### 3.3 MS data processing

**FIGURE 4:**
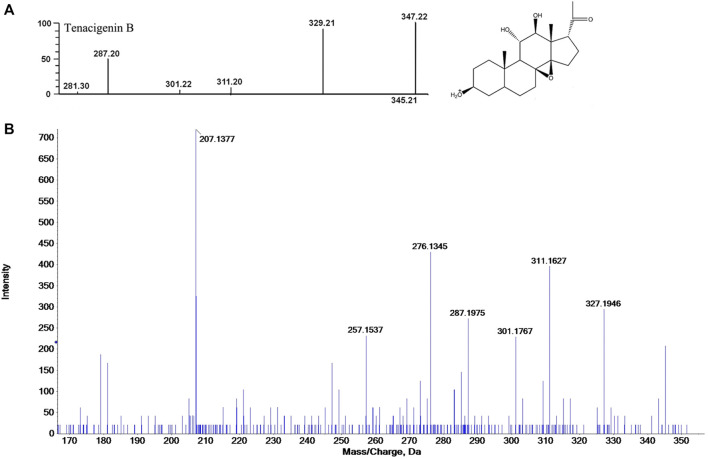
Tentative identification of metabolites, taking Tenacigenin B as an example. **(A)** Fragmentation pattern by data review. **(B)** Confirmation of the identification by MS^E^ spectra and putative fragmentation pathway of Tenacigenin B.

In the past decade, there have been more than 100 compounds of C_21_ steroidal saponins isolated from *Marsdenia tenacissima*. The MS database of chemical constituents of *Marsdenia tenacissima* was established after a literature review (Chen et al., 2008). In this study, Peakview (version 2.1) software was used for identification by comparing it with the previously established MS database. MS data processing, including peak picking, retention time, charge-mass ratio, and ion fragments, were used as the criteria for identification. The process of tentatively identifying a metabolite is shown in Figure 4, taking Tenacigenin B as an example, the MS spectra recorded in the database show that the ion fragments of Tenacigenin B including 345, 329, 311, 301, 287 (Zhao et al., 2016) (Figure 4A). Figure 4B shows that fragmentation of ions is the same as MS database, supporting the identification of Tenacigenin B.

**FIGURE 5:**
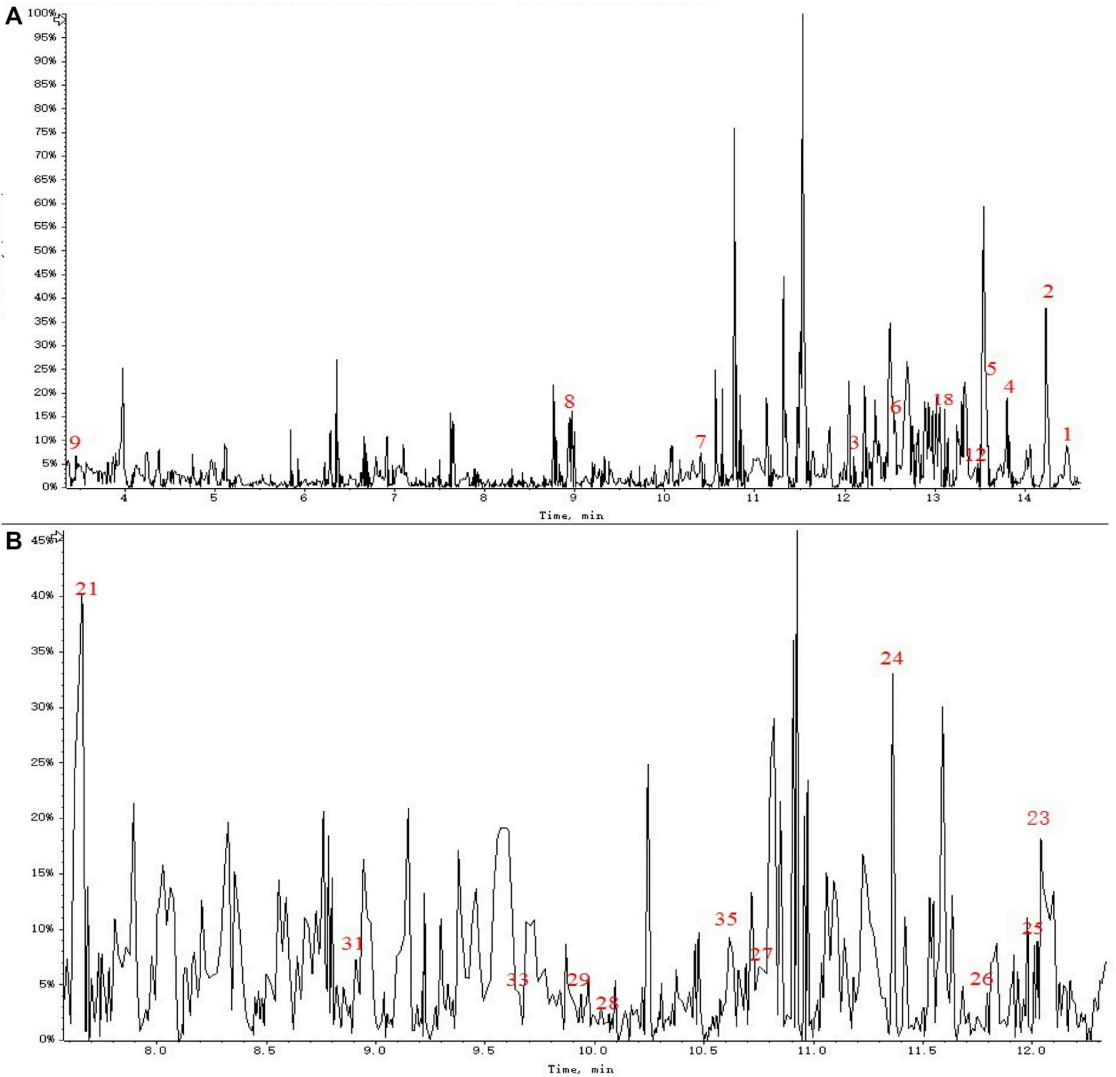
**(A)** TIC chromatogram of MGF sample (pos). **(B)** TIC of MT sample (neg).

**FIGURE 6:**
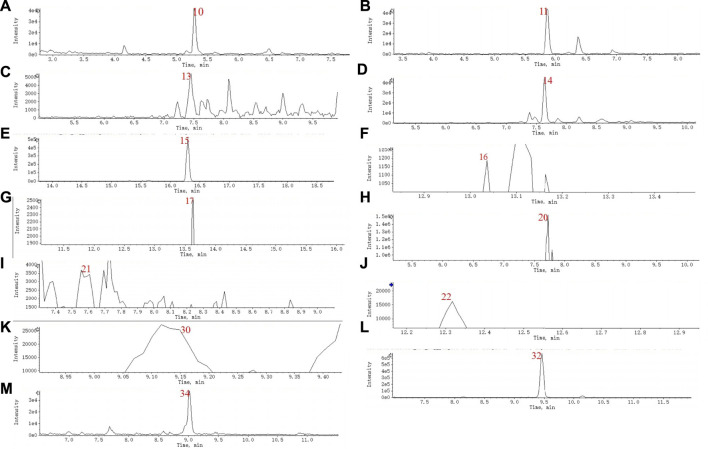
**(A–M)** XIC of the NO.10, 11, 13, 14, 15, 16, 17, 20, 21, 22, 30, 34 and 32.

A total of 36 principal components were identified from the three samples in Table 1, including 31 compounds C_21_ steroid saponins from *Marsdenia tenacissima*, and 5 compounds were *Ganoderma lucidum* components. The chromatogram of these components are shown in Figures 5A,B and Figures 6A–M. The distribution of these 36 components in three samples was compared in Table 1. Among them, C_21_ steroidal saponin has antitumor activity, Tenacigenin B has an antitumor effect in Lymphoma *in vitro* and *in vivo* (Xie et al., 2019). Ganoderic acid DM showed significant inhibitory effect on A549 lung cancer cells (Xia et al., 2020). Tenacissoside G, Tenacissimoside H and Tenacissoside I possess promising potential in anti-inflammatory, cancer chemopreventive, and neuroprotective medicines dietary or supplements (Liu et al., 2008; Wang et al., 2021). Ganoderic acids protect against alcoholic liver injury and modulate intestinal microbiota in mice with excessive alcohol intake (Guo et al., 2022).

**TABLE 1:** Tentatively identified major metabolites of *Marsdenia tenacissima* (MT), *Ganoderma lucidum* (GL) and *Marsdenia tenacissima* with *Ganoderma lucidum* co-fermentation (MGF).

NO	RT_EM	Tentative identification	Adducts	Formula	Mass error (ppm)	Ocurence		
						GL	MGF	MT
1	14.477_553.3134 m/z	11α-O-2-Methylbutyryl-12β-O-2-benzoyltenacigeninB	[M + H]+	C_33_H_44_O_7_	−4.69		√	√
2	14.269_531.3321 m/z	11α-O-2-Methylbutyryl-12β-O-2-tigloyltenacigeninB	[M + H]+	C_31_H_44_O_7_	−3.01		√	√
3	12.007_529.3150 m/z	11α, 12β-Di-O-tigloyltenacigeninB	[M + H]+	C_31_H_44_O_7_	−2.45		√	√
4	13.800_551.2981 m/z	11α-O-Tigloyl-12β-O-benzoyl-marsdenin	[M + H]+	C_33_H_42_O_7_	−3.99		√	√
5	13.566_529.3147 m/z	11α, 12β-Di-O-tigloyltenacigeninB	[M + H]+	C_31_H_44_O_7_	−2.45		√	√
6	12.561_511.2696 m/z	11α-O-Tigloyl-12β-O-benzoyl-marsdenin	[M + H]+	C_30_H_38_O_7_	−3.71		√	√
7	10.412_489.2840 m/z	11α-O-Tigloyl-12β-O-acetyltenacigenin B	[M + H]+	C_28_H_40_O_7_	−1.43		√	√
8	8.954_447.2730 m/z	11α-O-Tigloyl-17β-tenacigenin B	[M + H]+	C_26_H_38_O_6_	−2.45		√	√
9	3.463_383.2418 m/z	Tenacigenin C	[M + H]+	C_26_H_38_O_7_	−2.34		√	√
10	5.302_363.2174 m/z	Tenacigenin A	[M-H]-	C_21_H_32_O_5_	−0.55		√	√
11	5.864_363.2166 m/z	Tenacigenin B	[M-H]-	C_21_H_32_O_6_	−2.75		√	√
12	13.467_837.4424 m/z	Tenacissoside I	[M + Na]+	C_44_H_62_O_14_	−0.95		√	√
13	7.411_515.2973 m/z	Ganoderenic acid C	[M-H]-	C_30_H_44_O_7_	−1.23	√	√	
14	7.647_517.3132 m/z	Ganoderic Acid C2	[M-H]-	C_30_H_46_O_7_	−2.34	√	√	
15	16.286_467.3145 m/z	Ganoderic acid DM	[M-H]-	C_30_H_44_O_4_	−0.91	√	√	
16	13.012_571.2836 m/z	Ganoderic acid H	[M-H]-	C_32_H_44_O_9_	0.13	√	√	
17	13.623_437.3455 m/z	Ganoderol A	[M-H]-	C_30_H_46_O_2_	−0.22	√	√	
18	13.071_815.4134 m/z	Tenacissoside G	[M + Na]+	C_42_H_64_O_14_	−1.34		√	√
19	9.425_713.3759 m/z	Marsdenoside F	[M + HCOO]-	C_35_H_56_O_12_	0.98			√
20	7.688_829.4216 m/z	Tenacissimoside H	[M-H]-	C_47_H_76_O_22_	−0.12		√	√
21	7.636_845.4080 m/z	Tenacissoside L	[M-H]-	C_41_H_66_O_48_	−5.01			√
22	12.340_775.4198 m/z	Marsdenoside D	[M + Na]+	C_40_H_64_O_13_	−5.28			√
23	12.049_843.4146 m/z	Marsdenoside J	[M-H]-	C_45_H_64_O_15_	−3.07			√
24	11.381_953.4735 m/z	Tenacissoside A	[M-H]-	C_45_H_70_O_14_	−1.78			√
25	11.993_955.4019 m/z	Marsdenoside H	[M-H]-	C_48_H_76_O_19_	1.15			√
26	11.790_953.4748 m/z	Tenacissoside A or Marstenacisside A2	[M-H]-	C_48_H_74_O_19_	−0.41			√
27	10.750_969.4740 m/z	Marstenacisside A3	[M-H]-	C_48_H_74_O_20_	4.02			√
28	10.078_993.4696 m/z	Ag = 3, R1/R2 = Bz/Ac, R3 = Neo	[M-H]-	C_50_H_74_O_20_	−0.50			√
29	9.944_913.4430 m/z	Tenacigenoside K	[M-H]-	C_47_H_70_O_19_	−0.98			√
30	9.143_975.4765 m/z	Ag = 6, R1/R2 = mBu/H, R3 = Neo	[M + HCOO]-	C_36_H_74_O_19_	−4.20			√
31	8.90_971.4846 m/z	Ag = 3, R1/R2 = Ac/Tig, R3 = Neo	[M-H]-	C_48_H_76_O_20_	−1.13			√
32	9.467_713.3760 m/z	Tenacissoside F	[M + HCOO]-	C_35_H_56_O_12_	0.84			√
33	9.631_971.4853 m/z	Ag = 3, R1/R2 = Ac/Tig, R3 = Neo	[M-H]-	C_48_H_76_O_20_	−0.41			√
34	9.011_729.3700 m/z	Ag = 6, R1 = R2 = H, R3 = Pac	[M + HCOO]-	C_35_H_56_O_13_	−0.68			√
35	10.619_973.4646 m/z	Ag = 6, R1/R2 = Tig/H, R3 = Neo	[M-H]-	C_46_H72O_19_	−0.40			√

RT-EM: Retention time-exact mass.

### 3.4 Non-targeted metabolomics analysis

**FIGURE 7:**
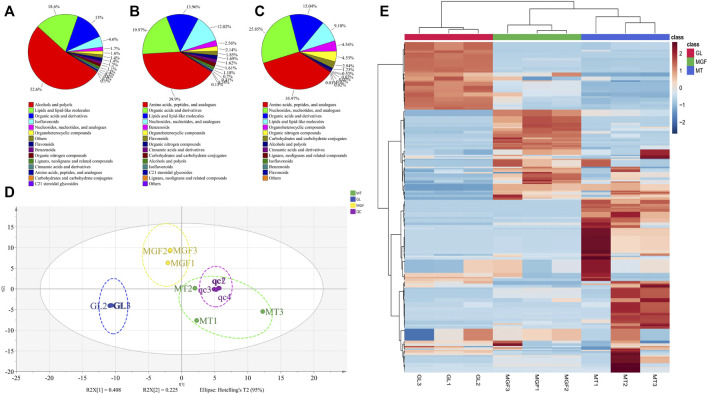
Depicting the biochemical categories of the differential metabolites identified. **(A)**:MT, **(B)**:MGF, **(C)**:GL. **(D)**: A score plot of the PCA demonstrating distinct metabolomes for MT, GL and MGF. **(E)**: Cluster analysis of metabolites from samples of MT, GL and MGF. The color indicates the level of accumulation of each metabolite, from low (blue) to high (red). The score represents the deviation from the mean by standard deviation units.

In order to better understand the fermentation fore-and-aft of *Ganoderma lucidum* and *Marsdenia tenacissima* what happened, we performed non targeted LC-MS/MS metabolomics for “MT”, “GL” and “MGF”, which represented *Marsdenia tenacissima* with *Ganoderma lucidum* co-fermentation, *Ganoderma lucidum* and *Marsdenia tenacissima*, respectively. In total, 249 metabolites were identified, which included a large number of metabolites likely to contribute to biotransformation during fermentation (17 amino acids, five carbohydrates, 35 organic acids) as well as other primary and secondary metabolites (Supplementary Table S3). These 249 metabolites were more than 15 different species (Figure 7) and the main proportion for three samples was Alcohols and polyols, Lipids and lipid-like molecules, Organic acids and derivatives, Flavonoids and Nucleosides, nucleotides and analogs. C_21_ steroidal saponins not only appeared in *Marsdenia tenacissima* (MT) but also *Marsdenia tenacissima* with *Ganoderma lucidum* co-fermentation (MGF) detected. The proportion C_21_ steroidal saponins in MT is 0.1% (Figure 7A), and the proportion of MGF is 0.41% (Figure 7B).

### 3.5 Principal component analysis and supervised OPLS-DA reveals differential metabolites in MT, GL, and MGF

To characterise the global metabolic differences between *Marsdenia tenacissima* (MT), *Ganoderma lucidum* (GL) and *Marsdenia tenacissima* with *Ganoderma lucidum* co-fermentation (MGF), a principal component analysis (PCA) was applied. Principal component analysis (PCA) is a multivariate statistical method to investigate the correlation between multiple variables. It studies how to reveal the internal structure of multiple variables through a few principal components and tries to recombine the original many with certain correlations into a new set of independent, comprehensive indicators to replace the original indicators (Ahsan et al., 2022). The statistical evaluation by PCA showed samples from *Marsdenia tenacissima* (MT), *Ganoderma lucidum (GL)* and *Marsdenia tenacissima* with *Ganoderma lucidum* co-fermentation (MGF) were located in different areas of the figure, which suggested that each sample have different metabolites (Figure 7D).

To eliminate the effects of quantity on pattern recognition, we applied a log10 transformation of peak areas for each metabolite and subsequently performed HCA to eliminate the effect of quantity on pattern recognition. HCA can eliminate the effect of quantitative differences, classify metabolites, classify metabolites with the same characteristics into the same category, and determine the degree of change of the metabolites in the same group. This analysis revealed the changes between GL, MGF and MT. The heatmap shows this analysis revealed three distinct groups associated with “MT”, “GL” and “MGF” respectively (Figure 7E). Different regions showed that the metabolites changed greatly before and after fermentation.

**FIGURE 8:**
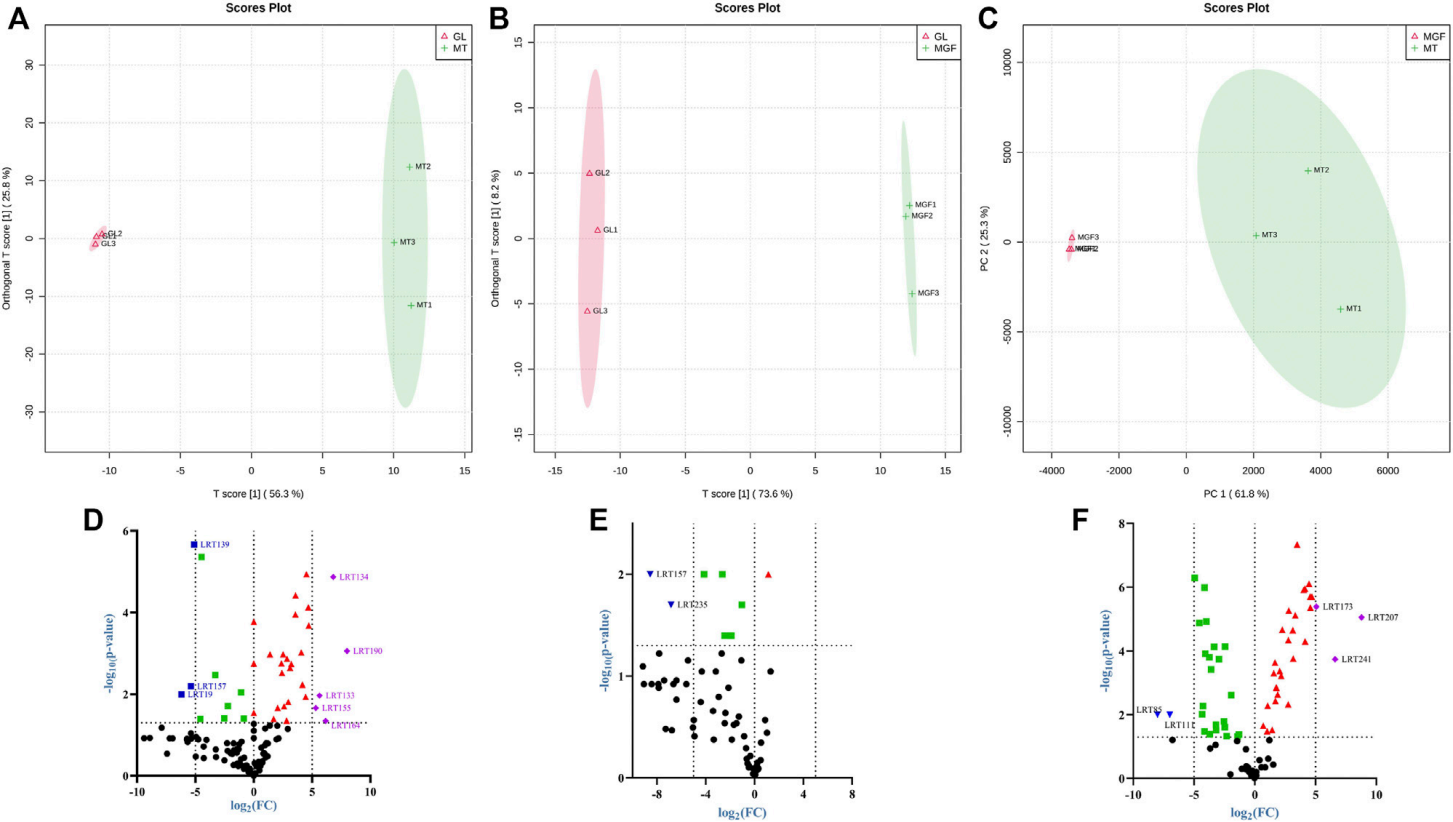
OPLS-DA score plot **(A–C)** for the OPLS-DA mode separating four types of MT, GL and MGF. Volcano plot of the 249 metabolites identified. **(D)** “MGF” compare to “MT”. **(E)** “MGF” compare to “GL”. **(F)** “GL” compare to “MT”.

In order to eliminate the noise information that is not related to classification and also to screen the credible metabolites that lead to classification differences, orthogonal partial least-squares discriminant analysis (OPLS-DA) is selected to filter the signals that are not related to classification, namely orthogonal signals, so as to obtain the OPLS-DA model (Zou et al., 2020). Figures 8A–C show that the score chart shows good separation between samples, indicating that there were significant differences between the metabolites from different samples. The characteristics of the OPLS-DA. Parameters *R* ^2^ and Q^2^ represent the interpretability and predictive ability of the model respectively, and the closer to one the better. Supplementary Table S5 indicated that the prediction ability of this model was good, and most of the data were simulated.

### 3.6 Analysis of differential metabolites

Among these metabolites, we compared the MT, MGF and GL samples and log_2_ (Fold Change) ≥ 5 or ≤ −5, *p* ≤ 0.05 were selected as the significantly different metabolites (Supplementary Table S4). The Volcano plot show differences up or downward trend in 249 metabolites among the three groups (Figures 8D–F).

### 3.7 Differences in primary metabolites between MT, GL and MGF

#### 3.7.1 Differences in organic acids between GL and MGF, MGF and MT

**FIGURE 9:**
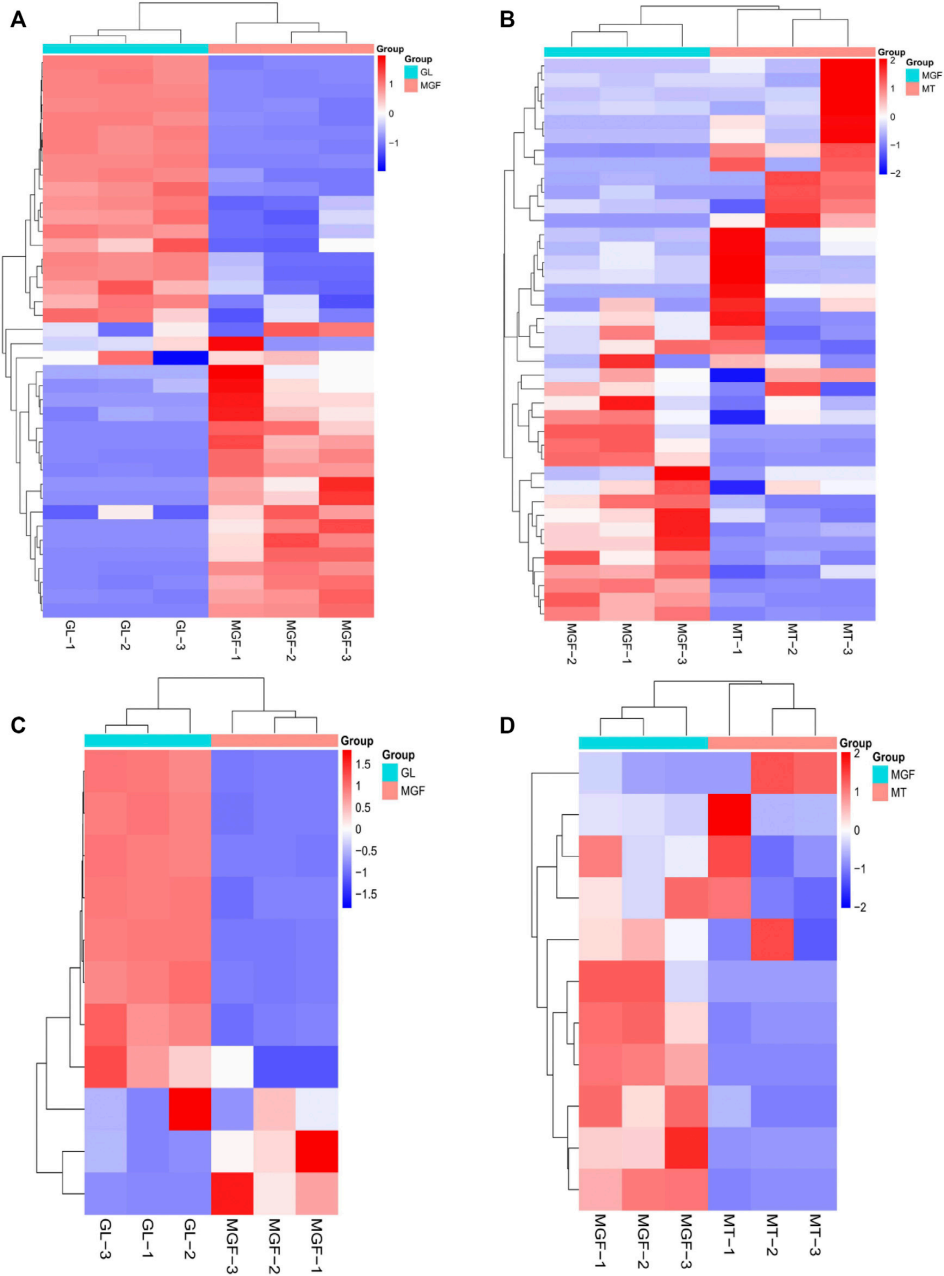
Thermograph of differential metabolites between MT and MGF. **(A)** Organic acids of GL compare MGF. **(B)** Organic acids of MGF compare MT. **(C)** Amino acids and derivatives of GL compare MGF. **(D)** Amino acids and derivatives of MGF compare MT.

Organic acids are the main source of three samples. Organic acids are mainly used as animal feed additives and antimicrobial agents for meat products. A total of 47 differential metabolites of organic acids were identified (Figures 9A,B). Nearly half of the organic acids were upregulated and another half were downregulated. The metabolites between GL with MGF (Supplementary Table S6), 25 organic acids such as 16a-Hydroxydehydrotrametenolic acid, caffeic acid cynarin, 4-Hydroxybenzoic acid and glycyrrhizic acid were significantly increased in MGF (Figure 9A). Madecassic acid, salvianolic acid C and isoferulic acid have first appeared in MGF. Compared MGF with MGF showed upregulation of 21 organic acids (Supplementary Table S6), vanillic acid, echinocystic acid, isoferulic acid, gallic acid and chlorogenic acid are significantly upregulated organic acids (Figure 9B). The cynarin of fermented products increased 600 folds compared with *Ganoderma lucidu*m and 60 folds compared with *Marsdenia tenacissima*. The cynarin can prevent cholesterol synthesis and low-density lipoprotein oxidation (Lattanzio et al., 2009). Chlorogenic acid (CGA) is a polyphenol which a proven ability to ameliorate some metabolic diseases through various pathways. CGA inhibited EGFR/PI3K/mTOR, HIF, VEGF pathways and MAPK/ERK pathway that may suppress tumor cell growth (Lukitasari et al., 2018).

#### 3.7.2 Differences in amino acids and their derivatives between GL and MGF, MGF and MT

Amino acids and their derivatives are distributed differently in the three samples. It can be seen from Figure 9C that compared with the GL group, only two kinds of amino acids in the MGF group were upregulated (Supplementary Table S6). Because when fermented to a certain extent, Ganoderma lucidum will produce decarboxylase, will decompose amino acids to produce polyamines. Most amino acids of MGF are upregulated compared with MT (9-D), because GL can decompose MT proteins to produce peptides, small peptides, free amino acids and other small molecules for their own growth. A total of 14 metabolic amino acids were detected, including 12 upregulated amino acids, mainly L-Valine, Leucine, Leucine, Serine and Isoorientin. Hydrolysis can gradually split protein molecules into smaller peptide units (Supplementary Table S6). With the increase of hydrolysis step by step, the solubility of the proteolytic enzyme increased gradually. The increased amino acids and peptides can be used as nutrients to be more conducive to the absorption and utilization of the human body and can also improve immunity and enhance the antibacterial and antioxidant activities.

### 3.8 Characteristic features of C_21_ steroidal saponins in *Marsdenia* tenacissima and *Marsdenia* tenacissima with Ganoderma lucidum co-fermentation

**FIGURE 10:**
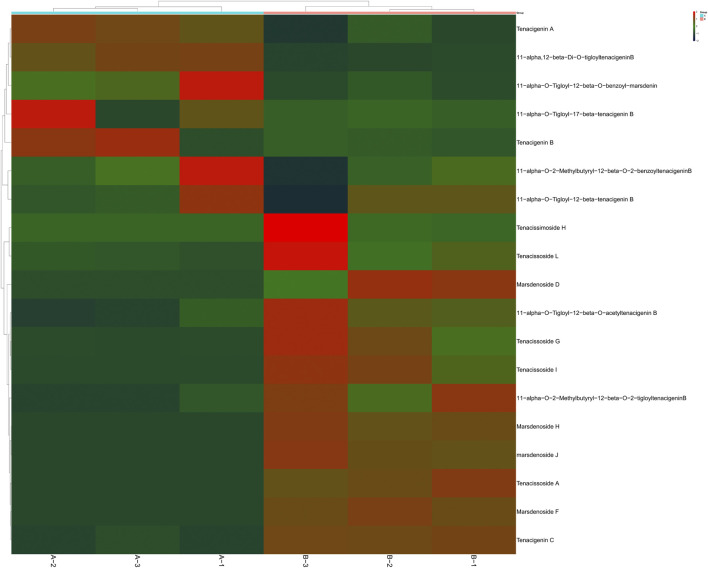
Heat-map of C_21_ steroidal glycosides in *Marsdenia tenacissima* (MT) and *Marsdenia tenacissima* with *Ganoderma lucidum* co-fermentation (MGF). **(A)**: MT **(B)**: MGF.

C_21_ steroidal glycosides in *Marsdenia tenacissima* have potent antitumor activity. It is clinically used for the treatment of liver cancer, gastric cancer and other malignant tumors with good curative effect (Liu et al., 2021). Over one hundred kinds of C_21_ steroidal glycosides were identified in *Marsdenia tenacissima*. Most C_21_ steroidal glycosides in *Marsdenia tenacissima* have the steroid mother nucleus of cyclopentanhydrophenanthrene. The common acyl groups at C-11α, C-12β and C-20 are acetyl (Ac), 2-methylbutyryl (mBu), 2-methylpropionyl (iBu), crotonyl (Tig), benzoyl (Bz) and 4-hydroxyphenylacetyl (HPA). The C-3β position was replaced by seven monoglycosides, such as Pac and Neo. The sugar chains are mainly composed of β-D-glucopyranose, α-D-glucopyranose, α-L-pyrano fucose, β-D-pyrano yellow MGFower caryophyllose, β-D-pyrano digitalis, β-D-pyrano caryophyllose, β-D-pyrano magnetoephedrine, and 6-deoxy-3-O-methyl-β-D-pyrano arabinose (Chen et al., 2008; Wang p.-l. et al., 2018). In this research, Table 1 shows 31 and 14 kinds of C_21_ steroidal glycosides in *Marsdenia tenacissima* were identified in *Marsdenia tenacissima* and *Marsdenia tenacissima* with *Ganoderma lucidum* co-fermentation. Seven kinds of tenacigenin, including 11α, 12β-Di-O-tigloyltenacigeninB, 11α-O-Tigloyl-12β-O-benzoyl-marsdenin, 11α-O-Tigloyl-12β-O-benzoyl-marsdenin 11α-O-Tigloyl-12β-O-acetyltenacigenin B, 11α-O-Tigloyl-17β-tenacigenin B, Tenacigenin A and Tenacigenin B were upregulated in *Marsdenia tenacissima* with *Ganoderma lucidum* co-fermentation (Figure 10A). 12 kinds of C_21_ steroidal glycosides in *Marsdenia tenacissima*, including Tenacissimoside H, Tenacissoside L, Marsdenoside D, 11α-O-Tigloyl-12β-O-acetyltenacigenin B, Tenacissoside G, Tenacissoside I, 11α-O-2-Methylbutyryl-12β-O-2-tigloyltenacigeninB, Marsdenoside H, Marsdenoside J, Marsdenoside F and Tenacigenin C were downregulated in *Marsdenia tenacissima* with *Ganoderma lucidum* co-fermentation (Figure 10B).

Table 1 shows that compared with *Marsdenia tenacissima* only tenacigenin was detected after fermentation by *Marsdenia tenacissima*, but C_21_ steroidal glycosides in *Marsdenia tenacissima* connected with glycosidic ligands were almost not detected. Previous research showed that Tenacissoside I, Tenacissoside G and Tenacissimoside H is the main component of saponins with multiple glycosidic ligands from *Marsdenia tenacissima*. We found that these three compounds were downregulated metabolites after fermentation.

### 3.9 Major metabolic pathways changes among Ganoderma lucidum and *Marsdenia* tenacissima with Ganoderma lucidum co-fermentation

**FIGURE 11:**
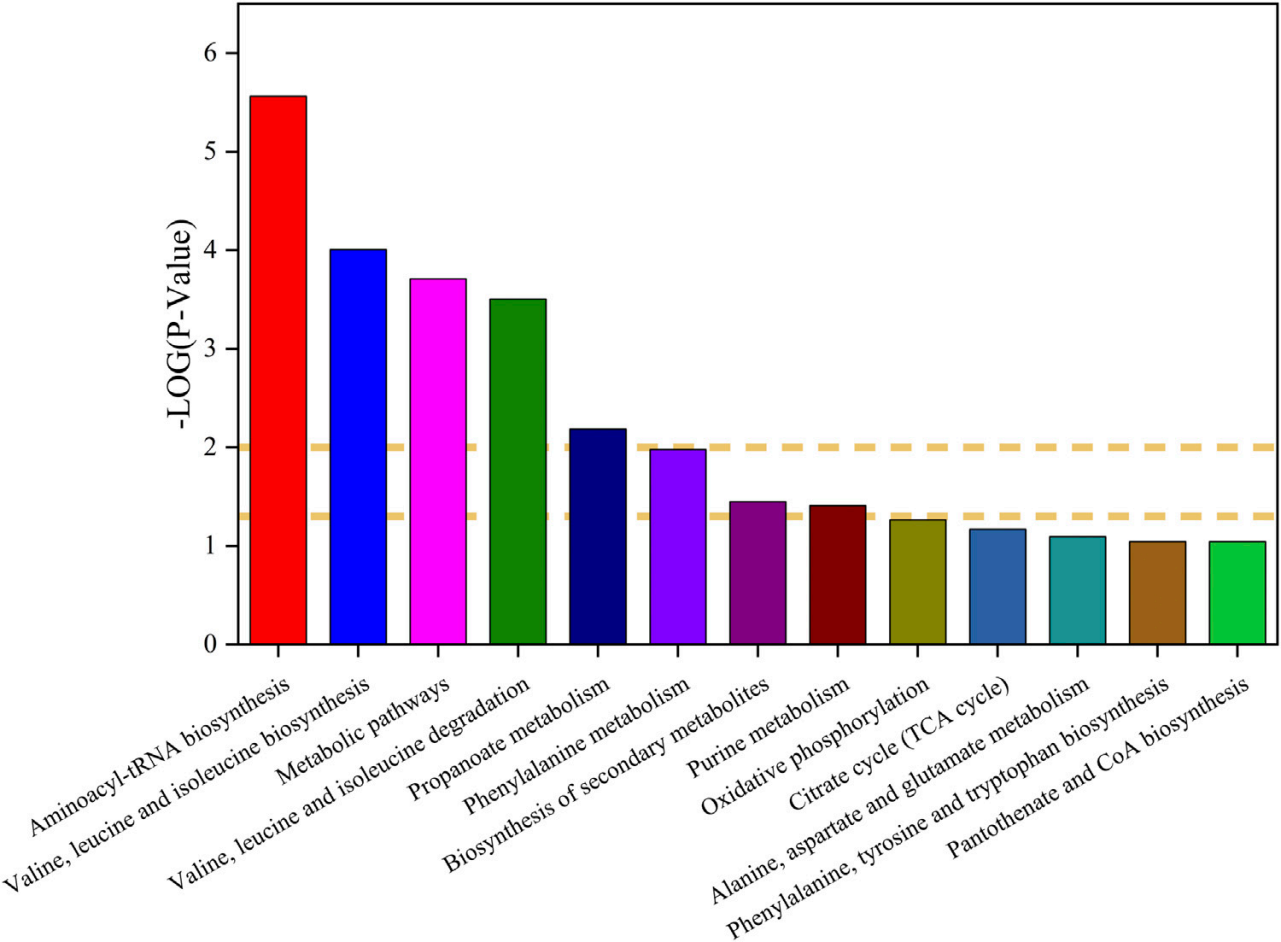
(I): KEGG pathway analysis of the differences between *Ganoderma lucidum* (GL) and *Marsdenia tenacissima* with *Ganoderma lucidum* co-fermentation (MGF).

The metabolites of *Marsdenia tenacissima* with *Ganoderma lucidu*m co-fermentation were compared with *Marsdenia tenacissima*, which were mapped to Kyoto Encyclopedia of Genes and Genomes (KEGG) database (http://www.genome.jp/kegg/) for metabolic pathway construction, The metabolic pathway map was plotted by the *p*-value of differential metabolites, where -log *p* > 1 was the up-regulated metabolic pathway and -log *p* < 1 was the down-regulated metabolic pathway (Ogata et al., 1999) (Figure 11). A total of 6 upregulated pathways and four significantly upregulated pathways, including Aminoacyl-tRNA biosynthesis, Valine, leucine and isoleucine biosynthesis, Metabolic pathways, Valine, leucine and isoleucine degradation, Propanoate metabolism. Downregulation of eight pathways, including alpha-Linolenic acid metabolism, Butanoate metabolism, Glyoxylate and dicarboxylate metabolism, Lysine degradation, *Glycine*, serine and threonine metabolism, Biosynthesis of unsaturated fatty acids, Tyrosine metabolism, Arginine and proline metabolism.

### 3.10 Comparison of antitumor efficacy after fermentation

**FIGURE 12:**
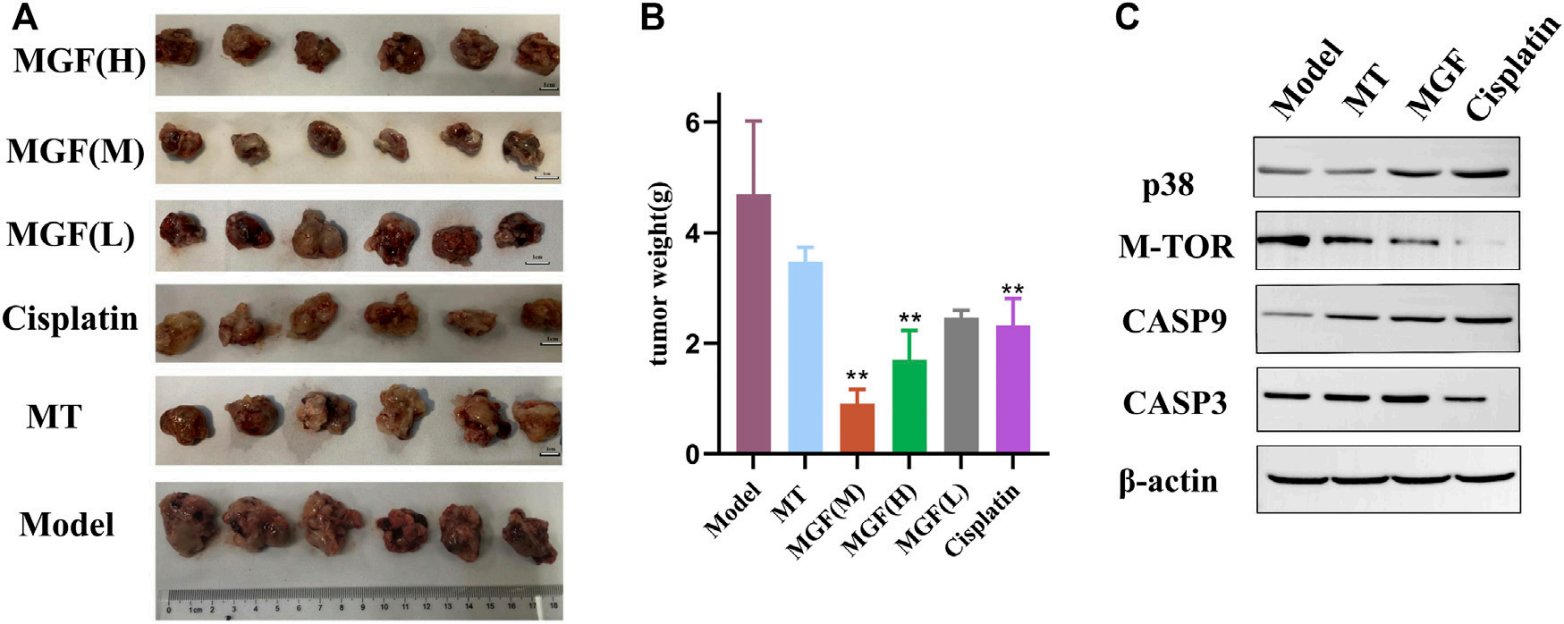
Anti-tumor effects *in vivo*. **(A)** Images of tumor tissue. **(B)** Mouse tumor weight. *p ＜ 0.05; **p ＜ 0.01 vs. MT group. **(C)** Western blot of several proteins.

In order to further explore the antitumor effect of *Marsdenia tenacissima* with *Ganoderma lucidum* co-fermentation compared with *Marsdenia tenacissima*, we chose the method of transplantation tumor in mice. The results reveal that in all mice, except the control groups, Tumor volume decreased and MGF (medium) group was the smallest (Figure 12A). The weight of the tumor in all groups was lower than that in the model group, and the MGF (medium) group was significantly lower than MT group, which also showed that the antitumor effect of MGF was significantly stronger than MT (Figure 12B).

### 3.11 Anti-lung cancer related proteins of MGF

In order to further clarify the molecular mechanism of the improvement of MGF anticancer effect, we next studied the growth and apoptosis related proteins. The results showed that the expression of MTOR in MGF group was lower than that in MT group (Figure 12C), indicating that MGF can inhibit the growth of cancer by inhibiting the growth of cancer cells in cancer tissues. In addition, we also examined caspase-3 and caspase-9 (Figure 12C). The results showed that the expressions of caspase-3 and caspase-9 in MGF group were higher than those in MT group, which suggested that MGF and MT achieved anti-cancer effect by inducing apoptosis, and the effect of MGF was better than MT.

## 4 Discussion

There have been many studies on the interaction between microorganisms and medicinal plants using non-target metabolomics technology. At present, there are many studies on the effects of microbial metabolism on the efficacy and ginsenoside changes of ginseng after biotransformation. Fungi itself contains a lot of glycoside hydrolases (Valadares et al., 2016). When the drug is used as the culture medium, the fungi use their own glycoside hydrolases to hydrolyze the glycosidic bonds connected to the chemical composition which belongs to drugs and the chain is broken as the carbon source for microbial growth (Beeson et al., 2015). The study on ginsenoside Rd in the residue of ginseng extract fermented by *Ganoderma lucidum* showed the changes of ginsenosides in fermentation products. They found that ginsenoside Rg1, Rd and compound K (CK) increased significantly after 30 days of fermentation (Chen et al., 2017). From our study, it can also be found that after *Ganoderma lucidum* fermentation, most of the saponins of C_21_ steroidal glycosides in *Marsdenia tenacissima* are Tenacigenin, even saponins with multiple glycosidic groups have not been detected (Table 1). It can be speculated that *Marsdenia tenacissima* uses its own enzyme system to hydrolyze the glycosidic bond of steroidal glycosides in *Marsdenia tenacissima*, and the broken monosaccharide or disaccharide is used for the growth of *Marsdenia tenacissima*. It can also be seen from the pathway of KEGG that the Metabolic pathway showed a significant upward trend in the MGF and GL (Figure 11).

In this study, not only the components of *Marsdenia tenacissima* such as Ganoderenic acid C, Ganoderic Acid C2, Ganoderic acid DM, Ganoderic acid H and Ganoderol A were detected, but also the saponins of *Marsdenia tenacissima* which include11α-O-Tigloyl-17β-tenacigenin B, Tenacigenin C, Tenacigenin A, Tenacigenin B,Tenacissoside I, Tenacissoside G and Tenacissimoside H were detected from the fermentation products. Ganoderic acid A can inhibit the release of histamine and enhance the functioning of digestive organs in human Additional pharmacological studies have shown that Ganoderic acid A can reduce blood fat, decrease blood pressure and regulate liver functions (Chen et al., 2017; Cao et al., 2020; Zhang et al., 2020). Previous studies have indicated that Ganoderic acid C2 possesses potential antihistamine, anti-tumor, anti-aging and cytotoxic properties (Liang et al., 2019; Li et al., 2021). Tenacigenin A is considered one of the main active ingredients. Previous results suggested that Tenacigenin A shows a good protective action on liver function of female mice with HCC and the effects may be associated with the apoptosis of hepatoma cells induced by Tenacigenin A (Chen et al., 2017). From Table 1 we can find a lot of anticancer chemical constituents from the fermentation products, so we can boldly speculate that the anticancer efficacy will be significantly enhanced when the *Marsdenia tenacissima* are fermented by *Marsdenia tenacissima*.

Here, we have conducted a comprehensive analysis before and after the fermentation of *Marsdenia tenacissima*. Through the non-targeted metabonomics technology, we can not only quantify the known components but also identify the components through the spectrum. A total of 249 metabolites were obtained and annotated. Compared with before and after *Marsdenia tenacissima* fermentation, the content of amino acids increased, which also increased the bioavailability of *Marsdenia tenacissima*. After Ganoderma lucidum fermentation, the organic acids were significantly increased, which not only improved the bitterness of the original medicine but also enhanced its anti-inflammatory and antibacterial activities. The upregulated *Marsdenia tenacissima* saponins detected in the fermented product also indicate that its anticancer effect will be enhanced.

We established a tumor model in mice to determine the efficacy of the fermented products. The results also showed that *Marsdenia tenacissima* with *Ganoderma lucidum* co-fermentation could effectively inhibit the growth of lung cancer, which also indicated that the anticancer effect of the original medicinal materials could be enhanced by biological transformation, providing relevant evidence for the development of new Chinese medicine. Caspase-9 and caspase-3 encodes a member of the cysteine-aspartic acid protease (caspase) family. These proteins is thought to play a central role in apoptosis and to be a tumor suppressor. Deguelin, a natural rotenoid, is among the class of bioactive metabolites from a diverse range of plants with potential antineoplastic effects in different cancer subtypes, deguelin has been reported to inhibit tumor growth *via* different signaling pathways, including caspase-3, caspase-8, and caspase-9 (Tuli et al., 2021). M-TOR is a member of the phosphoinositide 3 - kinase family of protein kinases, which regulates cell growth and cell proliferation, and is a key target gene in cancer treatment. Trillium tschonoskii Maxim. (TTM), a traditional Chinese medicine, possesses potent anti-tumor effect and polyphyllin VI (PPVI) was successfully isolated from TTM with guidance of the anti-proliferative effect in A549 cells, the mechanism study found that the activity of mTOR which regulates cell growth, proliferation and autophagy was significantly suppressed by PPVI (Teng et al., 2019).

The *Marsdenia tenacissima* with *Ganoderma lucidum* co-fermentation techniques have been examined, optimized and compared with regard to increase of fermentation products. The results showed that the fermentation time was the greatest impact on the experiment. With the RSM conditions, we can get more fermentation products. This work has provided strong support for the expansion of *Marsdenia tenacissima* with *Ganoderma lucidum* co-fermentation and increased economic benefits, offering a new direction for the development of anticancer drugs.

## 5 Conclusion

In summary, fermentation can not only improve the bitterness of traditional Chinese medicine but also improve nutrients and active substances, which are more easily accepted by people. The primary metabolites and secondary metabolites before and after fermentation were analyzed by non-targeted metabolomics technology, and It was found that the original complex macromolecular substances of *Marsdenia tenacissima* fermented by Ganoderma lucidum could be degraded into monosaccharide glycosides, amino acids, small molecular organic acids and other active substances, which was not only beneficial to the absorption and utilization of the human body but also improve its medicinal value. Macromolecule polyglycoside-linked C_21_ steroidal glycosides in *Marsdenia tenacissima* were hydrolyzed to small molecule saponins, especially Tenacigenin C, Tenacigenin A and Tenacigenin B, which was an important anticancer substance of *Marsdenia tenacissima*. The fermentation products were also significantly upregulated, which is worthy of attention. This study also showed that it was feasible to biotransform *Marsdenia* tenacissima by microbial fermentation. The optimization of the fermentation process can increase the yield of fermentation products and achieve economic maximization. hui, 2000.

## Data Availability

The original contributions presented in the study are included in the article/Supplementary Material, further inquiries can be directed to the corresponding author.
